# Ptychographic X-ray speckle tracking with multi-layer Laue lens systems^1^

**DOI:** 10.1107/S1600576720006925

**Published:** 2020-07-08

**Authors:** Andrew J. Morgan, Kevin T. Murray, Mauro Prasciolu, Holger Fleckenstein, Oleksandr Yefanov, Pablo Villanueva-Perez, Valerio Mariani, Martin Domaracky, Manuela Kuhn, Steve Aplin, Istvan Mohacsi, Marc Messerschmidt, Karolina Stachnik, Yang Du, Anja Burkhart, Alke Meents, Evgeny Nazaretski, Hanfei Yan, Xiaojing Huang, Yong S. Chu, Henry N. Chapman, Saša Bajt

**Affiliations:** a CFEL, Deutsches Elektronen-Synchrotron DESY, Notkestrasse 85, 22607 Hamburg, Germany; b DESY, Notkestrasse 85, 22607 Hamburg, Germany; c National Science Foundation BioXFEL Science and Technology Center, 700 Ellicott Street, Buffalo, NY 14203, USA; d National Synchrotron Light Source II, Brookhaven National Laboratory, Upton, NY 11973, USA; e The Hamburg Centre for Ultrafast Imaging, Luruper Chaussee 149, 22761 Hamburg, Germany; f Department of Physics, Universität Hamburg, Luruper Chaussee 149, 22761 Hamburg, Germany

**Keywords:** X-ray speckle tracking, ptychography, wavefront metrology, X-ray optics, multi-layer Laue lenses

## Abstract

The ever-increasing brightness of synchrotron radiation sources demands improved X-ray optics to utilize their capability for imaging and probing biological cells, nano-devices and functional matter on the nanometre scale with chemical sensitivity. Hard X-rays are ideal for high-resolution imaging and spectroscopic applications owing to their short wavelength, high penetrating power and chemical sensitivity. The penetrating power that makes X-rays useful for imaging also makes focusing them technologically challenging. Recent developments in layer deposition techniques have enabled the fabrication of a series of highly focusing X-ray lenses, known as wedged multi-layer Laue lenses. Improvements to the lens design and fabrication technique demand an accurate, robust, *in situ* and at-wavelength characterization method. To this end, a modified form of the speckle tracking wavefront metrology method has been developed. The ptychographic X-ray speckle tracking method is capable of operating with highly divergent wavefields. A useful by-product of this method is that it also provides high-resolution and aberration-free projection images of extended specimens. Three separate experiments using this method are reported, where the ray path angles have been resolved to within 4 nrad with an imaging resolution of 45 nm (full period). This method does not require a high degree of coherence, making it suitable for laboratory-based X-ray sources. Likewise, it is robust to errors in the registered sample positions, making it suitable for X-ray free-electron laser facilities, where beam-pointing fluctuations can be problematic for wavefront metrology.

## Abstract 2

Simultaneous wavefront metrology and sample projection imaging with multi-layer Laue lenses using the ptychographic X-ray speckle tracking technique is described. Results from three experiments are presented.

## 1. Introduction   

**Figure 1:**
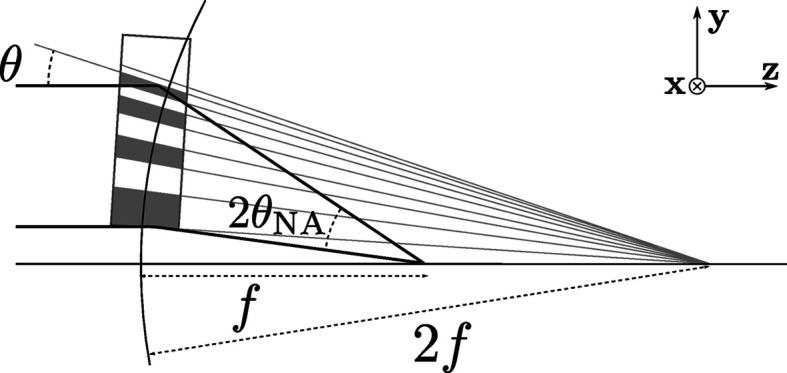
A wedged multi-layer Laue lens of focal length *f* is constructed from layers whose spacing follows the zone-plate condition. To achieve high efficiency the lens must be thick, in which case diffraction is a volume effect described by dynamical diffraction. In this case the layers should be tilted to locally obey Bragg’s law, which places them normal to a circle of radius 2*f*.

In 2015, Morgan *et al.* (2015 ▸) reported on the use of a lens for one-dimensional focusing of hard X-rays, with a photon energy of 22 keV. This lens was made by alternately depositing two materials with layer periods that follow the Fresnel zone-plate condition and then slicing the structure approximately perpendicular to the layers to the desired optical thickness. By varying the tilt of the layers throughout the stack, so that the Bragg and zone-plate conditions are simultaneously fulfilled for every layer, large focusing angles can be achieved with uniform efficiency. Such a structure is referred to as a wedged multi-layer Laue lens (MLL) (Yan *et al.*, 2014 ▸), which is fabricated by the use of a masked magnetron sputtering technique, and is schematically illustrated in Fig. 1 ▸.

Errors in the wavefront produced by the wedged MLL were characterized using a pseudo-one-dimensional ptychographic algorithm. This analysis revealed a defect in the lens that produced two distinct regions along the layer stack, each with a different focal length. Further studies revealed that the defect was caused by a transition in the material pair from amorphous to crystalline phase for layer periods of about 5.5 nm (Bajt *et al.*, 2018 ▸). By switching to a new material pair (tungsten carbide and silicon carbide) the phase transition could be avoided, allowing for a larger lens stack with greater focusing power. This illustrates the importance of wavefront metrology as a diagnostic tool for the iterative development of new optical elements.

Ptychography is a powerful tool for wavefront metrology, as it allows for the simultaneous recovery of the complex-valued wavefront produced by the lens and the complex-valued transmission function of the sample which is scanned across the wavefront, typically near the focal plane of the lens, with diffraction-limited resolution (Chapman, 1996 ▸; Rodenburg *et al.*, 2007 ▸; Thibault *et al.*, 2009 ▸). The high resolution is a result of the fact that ptychography often employs a fully coherent model^2^ for the wavefront propagation from the sample to the detector plane, with few approximations beyond paraxial illumination, a thin specimen and a high degree of coherence of the imaging system.

However, ptychography can present difficulties in its implementation, in part because the coherent model of the imaging system can be sensitive to errors in the estimated model parameters. It can also be computationally demanding to perform the required number of iterative steps in the reconstruction algorithm, which can be exacerbated by the large number of diffraction patterns in some ptychographic data sets. Furthermore, determination of the root cause of a failed reconstruction, for example, bad detector readings, sample stage jitter, X-ray source incoherence or algorithm parameters, can be difficult owing to the complicated relationship between the measured diffraction intensities and the recovered wavefronts. For example, although the wavefront reconstruction reported by Morgan *et al.* (2015 ▸) took only a few hours to complete, this calculation was preceded by many months of work identifying detector artefacts, exploring reconstruction parameters and checking the uniqueness of the output.

Since the work of Bérujon *et al.* (2012 ▸) and Morgan *et al.* (2012 ▸) (no relation to the current author), X-ray speckle tracking (XST) techniques have emerged as a viable tool for wavefront metrology applications. This method is based on near-field speckle-based imaging, where the 2D phase gradient of a wavefield can be recovered by tracking the displacement of localized ‘speckles’ between an image and a reference image produced in the projection hologram of an object with a random phase/absorption profile (random in the sense that the modulation of the beam by the object is both detailed and non-repeating over the relevant spatial frequencies of the image). Additionally, XST can be employed to measure the phase, absorption and ‘dark-field’ profile of an object’s transmission function. Thanks to the simple experimental setup, high angular sensitivity and compatibility with low-coherence sources, this method has since been actively developed for use in synchrotron and laboratory light sources; see Zdora (2018 ▸) for a recent review.

As part of an ongoing project to develop and improve the fabrication and performance of wedged MLLs for imaging (Prasciolu *et al.*, 2015 ▸; Murray *et al.*, 2019 ▸), we have developed a modified form of XST suitable for highly divergent illumination conditions (Morgan, Quiney *et al.*, 2020 ▸). This method, the ptychographic X-ray speckle tracking (PXST) technique, adopts an experimental geometry and iterative update algorithm similar to that employed in many ptychographic applications. Under ideal imaging conditions, the PXST method will not achieve the same (diffraction-limited) sample-imaging resolution or phase sensitivity that could be achieved via ptychographic approaches. However, we show that it is possible to recover images with large magnification factors, of around 2000 or more, and thus PXST can provide sufficiently high phase sensitivity and imaging resolution for many applications. On the basis of a pseudo-geometric approximation for the propagation of light from the sample exit surface to the detector plane, the source of errors in the recovered wavefronts can be localized to individual intensity measurements, leading to a more transparent and more easily diagnosed reconstruction process. We present PXST results from three separate experiments, each with a different sample, effective magnification and defocus distance.

## 2. Wavefront analysis   

**Figure 2:**
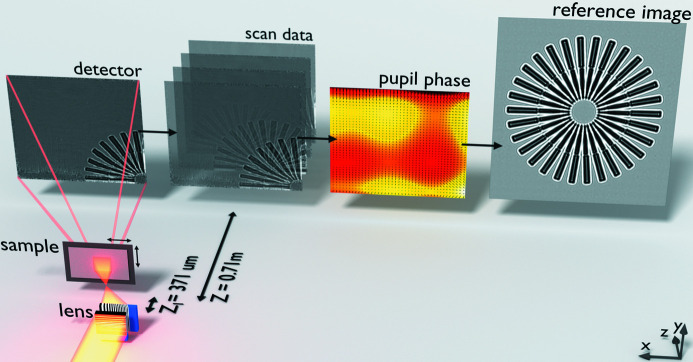
Illustration of the ptychographic XST method. The beamline illumination was focused (off-axis) in two dimensions by two crossed and wedged MLLs. The Siemens star sample was placed 371 µm downstream of the focal plane. Images were recorded on a pixel array detector 0.71 m downstream of the sample. The scan data consist of 20 × 20 shadow images, recorded as the sample was translated across the beam profile. The phase and reference image maps were refined iteratively.

The experiment setup and processing pipeline are roughly equivalent for each experiment, as illustrated in Fig. 2 ▸. In this configuration the sample was placed a distance *z*_1_ downstream of the 2D beam focus, which was formed using two crossed and wedged MLLs (one MLL to focus vertically and the other horizontally). The focal length of the lens closest to the focus was reduced by its distance from the other lens so that the focal points for the two MLLs meet in the same plane. A total of *N* images (*I*_*n*_) were then recorded on a detector placed a distance *z* downstream of the sample, as the sample was translated in a 2D grid pattern a distance Δ**x**_*n*_ in the *xy* plane (perpendicular to the optical axis for the *n*th image). If *z*_1_ is sufficiently large, then the images formed on the detector resemble shadow images of the sample, which are variously called Gabor or in-line holograms, near-field images, phase contrast images *etc.* depending on the specific application and properties of the sample (for the rest of this article we shall refer to such images simply as shadow images).

In the ideal case, for a thin sample, a lens system without any aberrations, ignoring diffraction from a lens aperture and for large *z*_1_, the lens will produce a spherical wavefront and it can be shown that the observed shadow image will be equivalent to a defocused and magnified image of the sample (*I*_ref_), such that 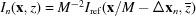, where the magnification factor *M* is given by (*z*_1_ + *z*)/*z*_1_ and the effective defocus 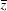 is given by *zz*_1_/(*z*_1_ + *z*). Morgan, Quiney *et al.* (2020 ▸) generalized this principle to incorporate the divergent illumination formed by a non-ideal lens system, so that 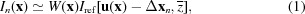
where *W*(**x**) is the ‘white-field image’, the intensity distribution measured on the detector without the presence of the sample. **u**(**x**) is a 2D vector field that captures both the average magnification of the image (due to the global phase curvature of the illumination) and the geometric distortions (arising from the finite aperture and lens aberrations) in the shadow image, given by 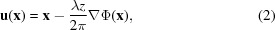
where λ is the wavelength of the radiation, ∇ = (∂/∂*x*, ∂/∂*y*) is the transverse gradient operator and Φ is the phase of the wavefield produced by the lens system in the detector plane (in the absence of the sample).

Using equations (1) and (2) and the set of shadow images (*I*_*n*_), the wavefront formed by the two MLLs in the detector plane, given by the phase (Φ) and intensity (*W*), as well as the undistorted, magnified and defocused image of the sample, which we call the ‘reference’ *I*_ref_, was recovered by tracking the local displacement of features formed in each of the shadow images according to the recipe described by Morgan, Quiney *et al.* (2020 ▸) using a speckle tracking software package (https://www.github.com/andyofmelbourne/speckle-tracking; Morgan, Murray *et al.*, 2020 ▸). In this method, initial estimates for ∇Φ, *I*_ref_ and Δ**x** are iteratively refined until the sum squared error between the measurements and the forward model [given by the forward model in equation (1)] is minimized. The analysis presented here can be replicated by following the tutorial sections on the software web site. The parameters for each experiment are summarized in Table 1 ▸. In the bottom row of this table, we provide a link to the experiment data on the CXIDB for each of these experiments (Maia, 2012 ▸).

### 2.1. Image reconstruction with the example of the Siemens star sample   

**Figure 3:**
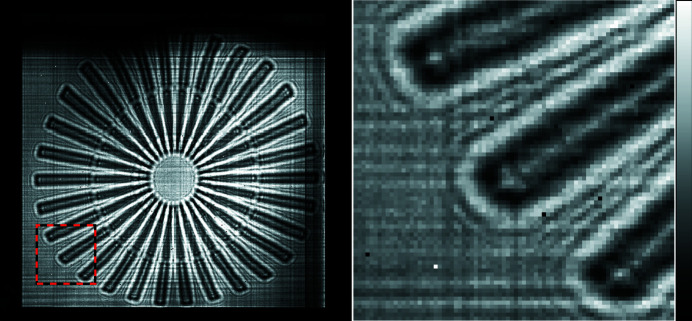
(Left) Raw detector image of the Siemens star shadow (480 × 438 pixels). The unfocused beam was blocked by a beam stop placed beyond the bottom left of this figure. The dashed red and black box outlines the region shown on the right at a higher image magnification. The linear colour scale is displayed on the far right and ranges from 0 (black) to 3000 (white) photon counts.

For this experiment, shadow images of a Siemens star test sample were recorded at the NSLS-II HXN beamline (Nazaretski *et al.*, 2014 ▸, 2017 ▸). Fig. 3 ▸ (left) shows one of the 400 shadow images recorded as part of the scan. To achieve a 2D focus we would ideally use two MLLs, one to focus vertically and the other horizontally, that are optimized for the same photon energy. In this experiment, however, we had one lens that was optimal at 16.7 keV and another at 16.9 keV. We decided to operate at 16.9 keV. Because of this mismatch of 0.2 keV, the vertically focusing MLL does not focus X-rays with uniform efficiency across the entire physical aperture. This results in the tapered fall-off in diffraction intensity near the top of the figure, corresponding to higher diffraction angles; the optical axis is located beyond the bottom left of the figure. The horizontally focusing MLL provided an X-ray focus with near perfect uniformity across the entire pupil region along the horizontal direction. In addition to scattering from the sample and the faint cross-hatch pattern (which we speculate are due to small local variations in the layer periods), there are also intensity variations across the image caused by the non-uniform illumination incident on the MLL lens system from up-stream optical elements.

The Siemens star test sample has a total diameter of 10 µm and consists of 30 radial ‘spokes’ with circular cuts at two radial positions. It is constructed from gold with a projected thickness in the range 0.5 to 1 µm. The ‘spoke’ tip, facing the centre of the star, has a width of 100 nm. In order to avoid speckle registration errors that would arise when translating features at different camera lengths across the field of view, for example, features at the top and bottom surface of the sample, the projected thickness of the sample (Δ*z*) should not be much greater than half the ratio of the demagnified pixel size (δ) to the numerical aperture of the lens system (NA): Δ*z* < δ/(2NA). In the present case, δ ≃ 30 nm and NA ≃ 0.017, leading to the condition Δ*z* < 0.9 µm, and so the Siemens star’s projected thickness is about the tolerable limit of this method for the current lens system and magnification factor. Similarly, the plane of translation of the sample must be nearly parallel to the plane transverse to the optical axis, such that ϕ < δ/FOV, where FOV is the field of view, or the side length of the footprint of the beam on the sample. In the present case, this limits the tilt angle to less than 24 mrad (to the best of our knowledge ϕ = 0 during this experiment).

The geometry of the Siemens star helps to visualize the effect of the low-order aberrations in the lens system on the observed shadow images. These aberrations led to low-spatial-frequency geometric distortions that break the approximate circular symmetry of the image, which is evident in Fig. 1 ▸ (left). To the right we show a magnified view of the region of interest. Here, we can observe approximately three Fresnel fringes generated by the sharp outer edges of the Siemens star spokes. This is the same fringe structure one would observe by illuminating the sample with plane wave illumination and recording an image on a detector placed a distance 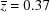
 m downstream of the object and magnified by a factor of *M* = 1917. The effective Fresnel number is then given by 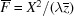, where *X* is the full-period spatial frequency of a feature in the sample. In the present case we have 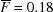, corresponding to the smallest width of Siemens star spoke, *X* = 100 nm.

The white-field image (*W*) was set to the median value at each pixel on the detector over the 400 measurements. A more direct approach would have been to record an image after completely removing the sample from the incident wavefield. We found, however, that the former strategy led to superior results. We speculate that this is due to low-frequency temporal drifts in either the positioning or the upstream illumination of the MLL system, leading to small variations in the intensity profile of the beam. Naturally, these drifts also occur during the acquisition time of the data set and could limit the viability of this method in cases where the duration of the experiment far exceeds the duration of stability for the imaging system.

The initial estimate for the gradient of the wavefield in the detector plane (∇Φ) was set to 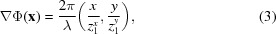
where 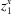 and 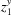 are the distances between the sample plane and the horizontal and vertical focal planes of the lens system, respectively. Note that for an astigmatic lens system, 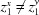. Estimates for 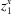 and 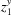 were obtained, in turn, by fitting a set of parameters in a forward model for the power spectrum of the data, obtained by summing the mod square of the Fourier transform of each image. The Fresnel fringes present in each image produce a nearly circular ring pattern in the cumulative power spectrum, known as ‘Thon rings’, where the shape and spacing of the rings provide estimates for defocus and astigmatism. This algorithm was adapted from the program *CTFFIND4* (Rohou & Grigorieff, 2015 ▸), which was developed for use on cryo-electron microscopy micrographs.

**Figure 4:**
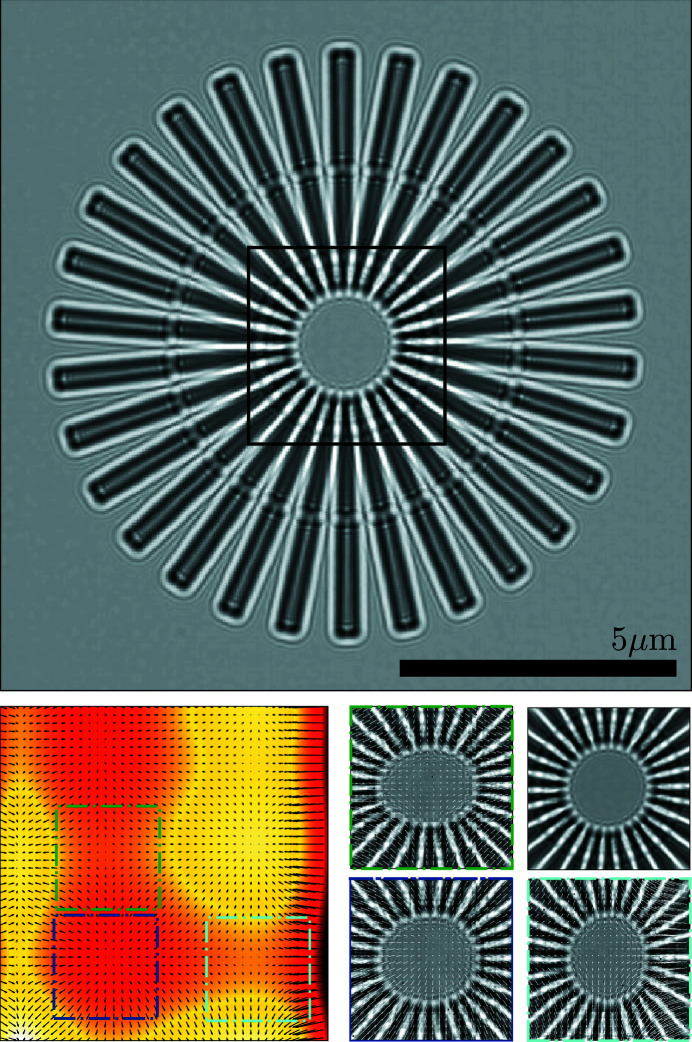
(Top) Reference image (*I*_ref_) of the Siemens star test sample. (Bottom left) Phase profile of the pupil function Φ (colour scale), overlayed with a quiver plot of the retrieved phase gradient ∇Φ vector field (scaled to pixel units). (Bottom right) Four views of the central region of the Siemens star. In the top right is the undistorted view (as outlined in black in the top panel). The remaining three panels show this feature as it appears in different locations on the detector array [after division by *W*(**x**)] corresponding to the regions indicated by like-coloured outlines in the left panel.

**Figure 5:**
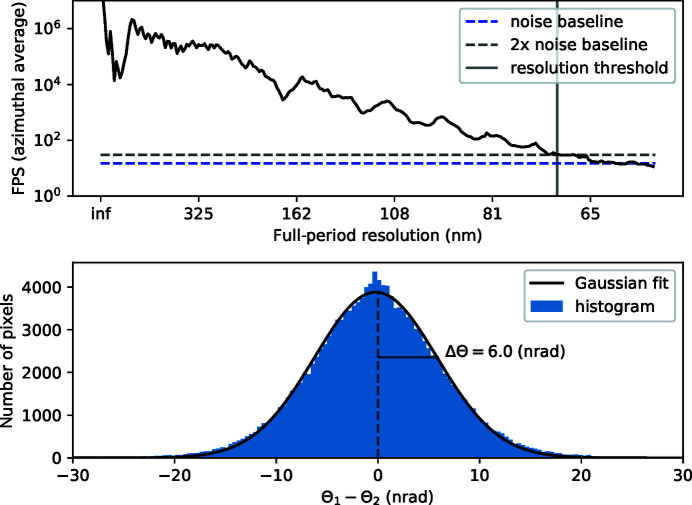
(Top) The azimuthal average of the Fourier power spectrum of the recovered reference image of the Siemens star sample. The FPS is obtained by taking the mod square of the Fourier transform of *I*_ref_. The blue dashed line shows the noise floor, which was estimated by taking the average of the FPS over the last 30 values. The resolution cut-off (grey vertical line) is given by the resolution at which the FPS is equal to twice the noise floor (black dashed line). (Bottom) Histogram of the difference between the recovered wavefront angles (detector plane) from each of the split-1/2 data sets (blue bar chart). The solid black line shows the Gaussian model fit with a standard deviation of 6.0 nrad.

In the top panel of Fig. 4 ▸ we show the reconstructed reference of the sample (*I*_ref_). We note that this is not a real-space image of the sample but rather a magnified view of the defocused image. Correctly reconstructed, this reference image will be free of the geometric aberrations present in each of the measured images, and indeed, this appears to be the case here. The direct (real-space) imaging resolution is limited by the effective defocus distance, so that point-like features will produce overlapping spots at a separation distance less than 331 nm (Rayleigh criterion), rather than the de-magnified pixel size of 28 nm. This is the separation distance between the inner edges of the spokes of the Siemens star when the first minimum of the edge’s Fresnel fringes overlaps with the brighter zeroth-order maximum of the adjacent edge. Another measure of resolution is the Fourier power spectrum (FPS) cut-off frequency, which is given by the highest spatial frequencies in an image above the signal-to-noise level. The FPS is graphed in Fig. 5 ▸ (top panel), with the vertical black line indicating the full-period resolution of the image at 70 nm, or a half-period resolution of 35 nm, approximately 20% greater than the de-magnified pixel size.

**Figure 6:**
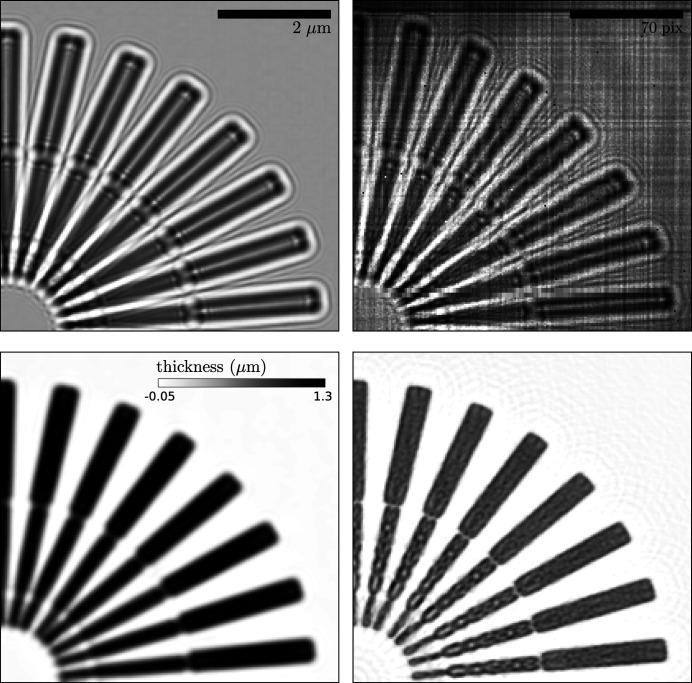
(Top left) Subregion of the reference reconstruction. The linear colour scale ranges from 0 (black) to 1.6 (white). (Top right) Subregion of image 250 in the data set, without any preprocessing. The linear colour scale ranges from 0 (black) to 4000 (white) photon counts. (Bottom left) TIE reconstruction of the same subregion as in the top-left panel. (Bottom right) CTF inverted reconstruction of the same region. The colour scale is the same as in the bottom-left panel.

In Fig. 6 ▸ we show two real-space reconstructions of the Siemens star’s projected mass (bottom left and right panels). For a sample constructed from a single material, with a constant density, and a linear approximation to Beer’s law, the projected mass is proportional to the thickness, or the height of the sample above the substrate. Both were recovered from the reference (top-left panel) and can be compared with a raw diffraction image, shown in the top-right panel. In the bottom row, we display the thickness profile recovered via the transport of intensity equation (TIE) and via contrast transfer function (CTF) inversion, in the left and right panels, respectively, using the *X-TRACT* software package (Gureyev *et al.*, 2011 ▸). We note that neither method is ideal in the present case: the TIE algorithm works best for large Fresnel numbers and the CTF inversion is ideal for weak phase objects. Nevertheless, the ends of the ‘spokes’ near the centre of the Siemens star, with a separation distance ∼158 nm, can clearly be distinguished in both images, which is an improvement on the direct (real-space) resolution of the reference.

The vector field **u**(**x**) − Δ**x**_*n*_ defines the mapping between each point in the *n*th image [*I*_*n*_(**x**)] and a point in the reference [see equation (1)]. The phase gradients were obtained from **u** via equation (2), using the formalism described by Morgan, Quiney *et al.* (2020 ▸), and are shown in the bottom-left panel of Fig. 4 ▸. Here we display the phase gradients, after removing the global shift and magnification factors, as a black quiver plot, scaled to pixel units. In order to further illustrate the effect of the geometric aberrations, beyond the overall magnification, caused by the phase gradients in the lens phase profile, we display a magnified view of the central region of the Siemens star (see the black box in the top panel of Fig. 4 ▸) as it appears in three different shadow images. The regions within the white field where this feature appears for each of the three images are illustrated by the coloured square outlines shown in the bottom-left panel. In the bottom-right panel we show the corresponding regions for each of these images. To increase the contrast, we have divided the images by the white-field image (*I*_*n*_/*W*). In the top-right sub-panel, we also show the same region of the recovered reference. Here one can clearly observe local variations in the degree of magnification, along the *x* and/or *y* axis, depending on the position of the sample within the incident wavefield.

**Figure 7:**
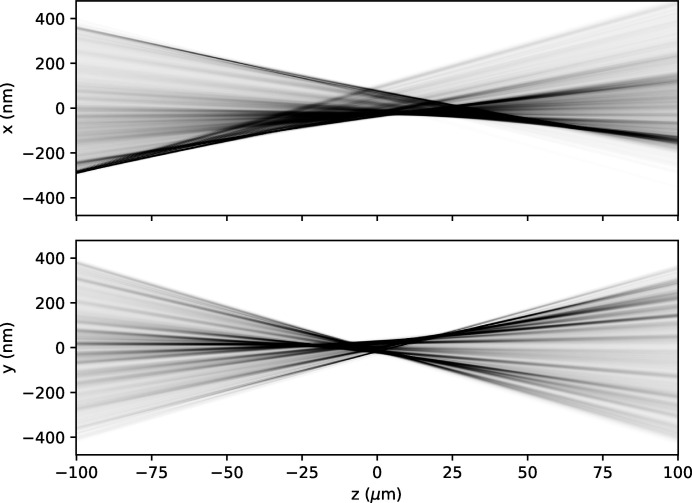
Projection of the wavefront intensity profile near the focal plane of the lens system, along the *x* axis (top) and the *y* axis (bottom). The linear colour scale ranges from 0 (white) to 1 (black) in arbitrary units.

In the bottom-left panel of Fig. 4 ▸ we show the residual phase profile of the MLL lens system (colour map). The residual phase profile is obtained after removing the constant, linear and quadratic components of the global phase profile, which correspond to an overall phase constant, a tilt term and the defocus aberrations, respectively. By removing these terms, it is possible to perceive the small deviations in the phase from an (ideal) quadratic profile. Armed with this phase profile, we could then numerically propagate the wavefield to the region near the focal plane of the lens, as shown in Fig. 7 ▸. These results were obtained after three iterations of the PXST update algorithm. For each iteration, we refined the initial estimates for the sample stage translations. The ‘irrotational constraint’ on the phase gradients was also enforced [see Section 5 of Morgan, Quiney *et al.* (2020 ▸)].

In Appendix *A* [App appa] we show a comparison of the recovered wavefront phase from a separate PXST experiment and a ptychographic experiment taken with the sample placed nearer to the focal plane. Both results show qualitative agreement; however, the root-mean-squared difference is much greater than we would predict if the ptychographic result is considered to be the ground truth.

The local angular distribution of the wavefront rays, in the plane of the detector, is given by 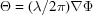. In the ideal case, the smallest resolvable angular deviation of a ray (the angular sensitivity) is given by ΔΘ = δ_pix_σ_det_/*zM*, where σ_det_ is the width of the point spread function of the detector (greater than or equal to the physical pixel size) and δ_pix_ is the fractional reduction in the effective pixel size due to numerical interpolation. In the present case, setting σ_det_ ≃ 55 m and δ_pix_ < 1, we have ΔΘ < 40 nrad.

In order to estimate the achieved angular resolution, we randomly assigned each pixel of each image to one of two data sets. Keeping the reconstructed reference map and sample stage positions from the original reconstruction, we then repeated the reconstruction of the phase gradients independently for each of the two data sets. This process is only possible because of the high degree of redundancy in the original data. A histogram of the difference between the two reconstructions, shown in the bottom panel of Fig. 5 ▸, provides an estimate for the underlying uncertainty in the recovered Θ values. The standard deviation of the difference Θ_1_ − Θ_2_ yields ΔΘ ≃ 6.0 nrad, which suggests a δ_pix_ value of less than 2/10. This shows that from the redundancy of data, caused by measurements at any given location in the wave with many positions of the object, one is able to interpolate angular deviations to a small fraction of a pixel. The angular distribution is related to the phase profile via 2D integration, 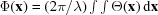, and propagating the uncertainties yields an estimate for the phase sensitivity of ΔΦ ≃ 0.065 rad (0.01 waves).

### 2.2. Diatom sample   

For this experiment, the biomineralized shell of a marine planktonic diatom was placed on a silicon nitride membrane and scanned across the wavefield 2.22 mm downstream of the lens focus. In contrast to the Siemens star experiment, the effective defocus and magnification (see Table 1 ▸) are such that only first-order Fresnel fringes are visible across the majority of the reference. For this reason we did not use the Thon rings to provide initial estimates for 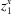 and 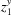. Instead, we set 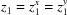 in equation (3) and chose the value of *z*_1_ which minimized the sum squared error after many trials over a range of *z*_1_ values. Errors in the initial estimates for *z*_1_, 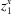 and 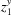 will lead to additional defocus aberrations in the recovered phase map, which can then be removed as needed. If these errors are too large, however, the algorithm may take many more iterations (or fail completely) to converge.

**Figure 8:**
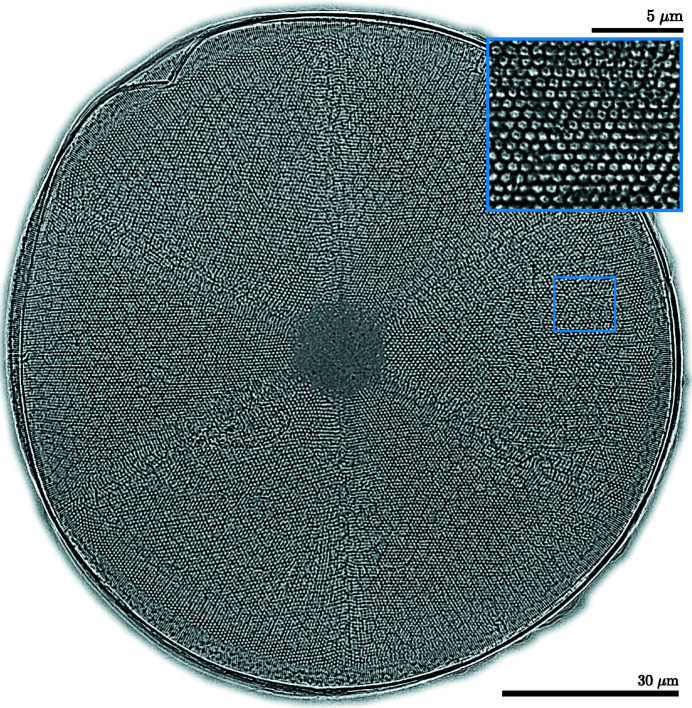
The diatom’s reference image. The linear greyscale colour map ranges from 0.92 (black) to 1.08 (white). The reconstructed area outside of the diatom’s region of interest has been masked. The demagnified pixel area is 93 × 93 nm. The field of view of the image is 122 × 120 µm (1320 × 1290 pixels). Fine details in the sub-structure of the diatom are visible in this phase-contrast projection image, which are otherwise obscured by the surface of the sample in scanning electron micrograph images. (Top right) Magnified image map of a subregion of the diatom. The field of view is 95 × 107 µm (414 × 466 pixels), with a demagnified pixel area of 24 × 24 nm. The small blue rectangle indicates the scale of the inset with respect to the larger image of the diatom.

In contrast to the previous experiment, only a fraction (roughly 1/9th) of the object is visible in the field of view for each image. The reference image is shown in Fig. 8 ▸, obtained after three iterations of the PXST algorithm.

This diatom was collected from the Antarctic sea and its shell is made from a complex network of nanostructured silica with an exceptional strength-to-weight ratio, despite being produced under low temperature and pressure conditions. The circular shell of the diatom is constructed from six azimuthal segments, which extend in a dome-like fashion out of the page for the orientation shown in Fig. 8 ▸. The boundary of these segments can be observed as six radial creases, extending from the edge of the inner circle to the outer rim of the sample. This sixfold symmetry is a motif that is repeated throughout the diatom structure: see for example the approximate hexagonal packing of the small ‘white dots’ with a diameter of about 5 µm. In another scan (discussed in the next section), taken with the sample closer to the focus, a more detailed view of these ‘white dots’ can be seen. This more magnified view of the diatom is displayed in the top-right corner of the figure, and one can see that these dots are themselves hexagonal in shape with what appear to be hollow depressions in the centre.

The estimated angular sensitivity for this reconstruction is 20 nrad, which is approximately 3.2 times greater than for the Siemens star reconstruction. This result is consistent with the corresponding decrease in the average magnification by a factor of 3.3, from 1917 (Siemens star) to 595 (diatom). The direct (real-space) imaging resolution was 410 nm (Rayleigh criterion), while the FPS cut-off frequency was 259 nm, with a half-period resolution of 130 nm, which is 40% greater than the de-magnified pixel size.

### 2.3. Diatom subregion   

For this experiment the sample was moved closer to the focal plane of the lens, from 2.22 mm in the previous section to 0.57 mm here. This corresponds to an increase in the magnification by a factor of 3.9, from 595 to 2308. As discussed by Morgan, Quiney *et al.* (2020 ▸), the upper limit to the magnification factor for this particular technique is governed by the smallest distance between the focal plane and the sample such that the diffraction remains in the near-field imaging regime. For larger magnification factors, with the sample closer to the focal plane, the rapidly oscillating phase and intensity of the illuminating wavefield lead to significant errors in the speckle tracking approximation of equation (1). Here, however, another difficulty was encountered, relating to the pseudo translational symmetry of the diatom structure at this magnification.

**Figure 9:**
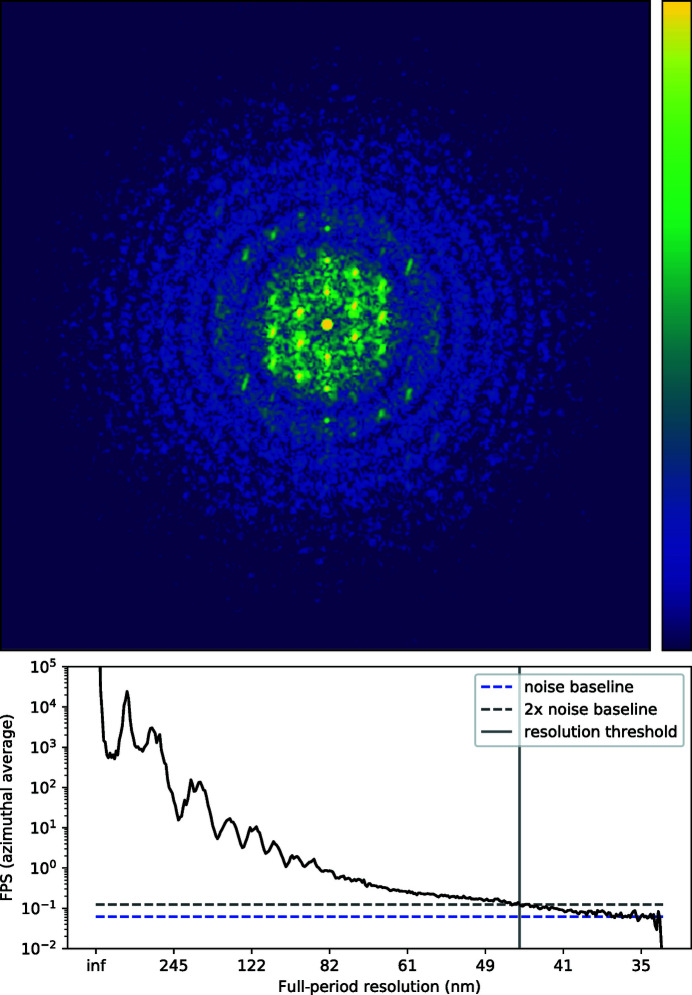
(Top) Image of the FPS of the diatom subregion. The full-period spatial frequency at the left edge of the image is 48 nm^−1^. To avoid artefacts from the sharp edges of the real-space image (as shown in the subpanel of Fig. 8 ▸) the FPS was filtered with a Gaussian window function with a standard deviation of 2.4 µm. Before display, the FPS was raised to the power 0.1, in order to reveal the Thon rings underneath the much stronger peaks from the hexagonal lattice. (Bottom) Azimuthal average of the FPS, with a cut-off frequency corresponding to a full-period resolution of 45 nm (half-period resolution of 22.5 nm, 5% smaller than the de-magnified pixel size).

The FPS of the reference, in the top panel of Fig. 9 ▸, shows an hexagonal array of points overlaid on top of the much weaker Thon rings, which (again) arise because the reference is a defocused image of the sample’s exit-surface wave. The locations of the peaks reveal the reciprocal lattice of the real-space structure, which is approximately hexagonal with a primitive lattice constant of ∼601 nm. This approximate translational symmetry is undesirable in PXST because of the possibility of miss-registering features between each recorded image and the reference by an amount equal to the lattice constant.

**Figure 10:**
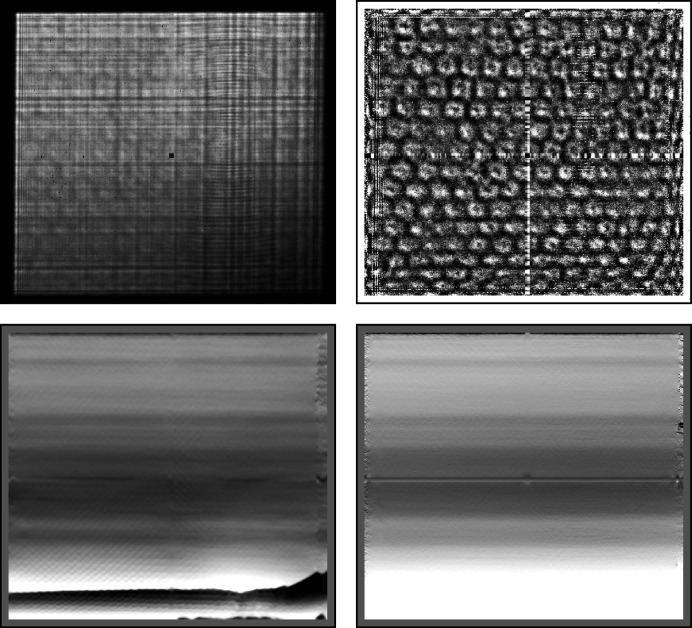
(Top left) Image 50 of the 121 recorded shadow images. This image spans diffraction angles of 15 × 17 mrad. The linear colour scale ranges from 0 (black) to 2000 (white) photon counts. (Top right) The same image divided by the white field (*W*); the colour scale ranges from 0.9 to 1.2. (Bottom left) The recovered pixel mapping between the recorded images and the reference image **u**(**x**) (in pixel units); the colour scale ranges from −10 (black) to 10 (white) pixel shifts. (Bottom right) The recovered pixel mapping when employing regularization during the reconstruction; the same colour scale as bottom left.

In the bottom-left panel of Fig. 10 ▸ we show a failed reconstruction of the pixel mapping between the recorded images (one of which is shown in the top-left panel) and the reference. At the bottom of the image one can see a horizontal step-like reduction in the mapping function from white to black, corresponding to a reduction of 20 pixels. When scaled to physical units, this drop corresponds exactly to the hexagonal lattice spacing of the diatom substructure. In order to overcome this problem, we chose to regularize the recovered pixel shifts by convolving them with a Gaussian kernel at each iteration. The standard deviation of this kernel was reduced linearly from 20 pixels to 0 pixels as the iterations progressed. In this way sharp deviations in the mapping function were prevented from forming early in the reconstruction process. The result of this regularization procedure is shown in the bottom-right panel of the figure, where the step-like artefact is no longer present in the reconstructed pixel mapping.

## 3. Discussion and conclusion   

In this article we have demonstrated the use of PXST on three experimental data sets. In each case, both the illuminating wavefront and a highly magnified, undistorted in-line hologram of the sample were recovered. The main benefit of PXST over other speckle tracking techniques, for example, the unified modulated pattern analysis (UMPA) approach of Zdora *et al.* (2017 ▸), the geometric flow algorithm of Paganin *et al.* (2018 ▸) and the original XST technique of Bérujon *et al.* (2012 ▸), is that it is able to deal with highly divergent illumination. This allows for comparatively large magnification factors (*e.g.* 2308 for the diatom subregion), which leads to a corresponding increase in the achievable ray angle sensitivity (3.4 nrad) and image resolution (45 nm full period). Conversely, PXST does not provide a direct (real-space) image of the sample’s phase, absorption or ‘dark-field’ profiles.

Another approach that is suitable for highly divergent illumination is the X-ray speckle scanning technique of Bérujon *et al.* (2012 ▸), which provides a phase sensitivity proportional to the step size of the sample translations. In PXST, however, the phase sensitivity does not depend on the step size, making it suitable for a broader range of experiment facilities.

With the high-NA, efficient, hard X-ray optics provided by the wedged MLLs used here, the footprint of the beam on the sample is greater for a fixed magnification factor than would otherwise be the case. This increases the throughput of the imaging method, by a factor proportional to the square of the increase in the NA.

We have also demonstrated that PXST does not require an additional diffuser in the beam path and we expect that a wide variety of samples could be used as a wavefront-sensing device – although a dense random object such as a diffuser should reduce the number of required images.

In future, we hope to develop the PXST algorithm for use in ‘cone-beam tomography’, a geometry where the illumination diverges significantly as it passes through the object.

## Supplementary Material

: https://doi.org/10.11577/1616246

: https://doi.org/10.11577/1616244

: https://doi.org/10.11577/1616245

## Figures and Tables

**Figure 11:**
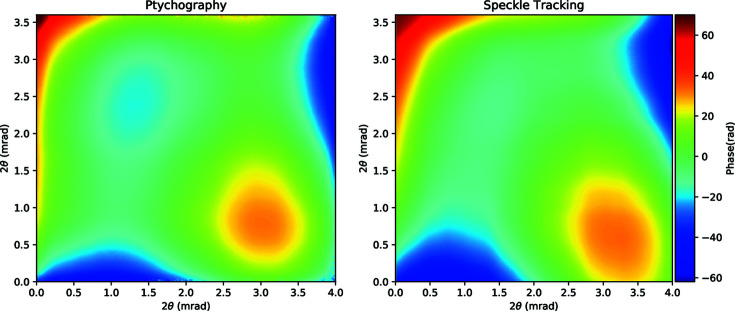
Phase of the recovered wavefronts via ptychography (left) and PXST (right). The ptychographic phase profile was unwrapped before display. The colour scale is in radian units.

**Table 1:** Parameters for the experiments

Sample	Siemens star	Diatom	Diatom subregion
Beamline	NSLS-II (HXN)	PETRA III (P11)	PETRA III (P11)
Energy (keV)	16.7	16.3	16.3
Focus–detector distance (m)	0.71	1.32	1.32
Focus-sample distance (mm)	0.371	2.22	0.55
Detector	Merlin	Lambda	Lambda
Detector grid (region of interest)	407 × 365	359 × 401	359 × 401
Physical pixel area (µm^2^)	55 × 55	55 × 55	55 × 55
Effective pixel area (nm^2^)	30 × 28	93 × 92	24 × 24
Average magnification	1917	595	2308
Effective defocus (mm)	0.37	2.21	0.57
Sample scan grid	20 × 20	11 × 11	11 × 11
Sample scan step size (µm)	0.63	10.00	0.20
Exposure time(s)	1	5	0.005
Iterations	3	3	20
Angular resolution (nrad)	6.3	20.0	3.4
CXIDB reference	https://www.cxidb.org/id-136.html	https://www.cxidb.org/id-134.html	https://www.cxidb.org/id-135.html

## Footnotes

2 Methods for dealing with partial coherence, which can be characterized by a few dominant modes, have been successfully developed for ptychography (Thibault & Menzel, 2013 ▸; Pelz *et al.*, 2014 ▸). Nevertheless, each mode is treated in a fully coherent way, consistent with the original (single-mode) ptychographic approach.

## Appendix A Comparison of the recovered wavefront via ptychography and PXST

In Fig. 11 ▸ we show the phase of the wavefront recovered via far-field ptychography (left) and PXST (right), from two independent data sets obtained at the European Synchrotron Radiation Facility.^3^ In both experiments, a Siemens star test sample was scanned in a 2D grid pattern across the wavefront, with a focus-to-sample distance of 0.13 m. For the ptychographic data set, the sample was scanned near the focal plane of the lens (*z*_1_ = 1.01 mm) and the angular extent of the diffraction extended well beyond that of the diverging illumination, *i.e.* outside of the holographic region. For the PXST data set, the sample was placed further from the focus (with *z*_1_ = 5.8 mm) and the diffraction was predominantly confined to the holographic region of the detector (in a 2 × 1.6 mrad angular window), consistent with the near-field scattering regime.

One advantage of PXST over ptychography is that the phase profile is not ‘wrapped’ onto the [−π, π) domain. This is useful in cases where the intent is for the recovered phases to inform a structural analysis of the lens system, such as the height of a mirror surface or the local period of bilayers in an MLL. In some cases, however, the phases recovered from ptychography can be ‘unwrapped’; for smooth phase profiles, continuity of the phases allows one to identify regions bounded by discontinuous 2π phase jumps. One can then add or subtract 2π to the phases in these regions as needed until the entire phase profile is smooth. This procedure was applied to unwrap the phases shown in the left panel.

The two phase profiles show qualitative agreement between the ptychographic and PXST algorithms. The root-mean-squared deviation is ∼5 rad, which is many orders of magnitude worse than the theoretically achievable phase sensitivity. Therefore, one or both of the reconstructions suffers from systematic artefacts in the recovered phases. This is a matter for further investigation.

## References

[bb1] Bajt, S., Prasciolu, M., Fleckenstein, H., Domaracký, M., Chapman, H. N., Morgan, A. J., Yefanov, O., Messerschmidt, M., Du, Y., Murray, K. T., Mariani, V., Kuhn, M., Aplin, S., Pande, K., Villanueva-Perez, P., Stachnik, K., Chen, J. P. J., Andrejczuk, A., Meents, A., Burkhardt, A., Pennicard, D., Huang, X., Yan, H., Nazaretski, E., Chu, Y. S. & Hamm, C. E. (2018). *Light Sci. Appl.* **7**, 17162. 10.1038/lsa.2017.162 PMC6060042 30839543

[bb2] Bérujon, S., Wang, H. & Sawhney, K. (2012). *Phys. Rev. A*, **86**, 063813.

[bb3] Chapman, H. N. (1996). *Ultramicroscopy*, **66**, 153. 10.1016/0304-3991(96)00003-4 8677527

[bb4] Gureyev, T. E., Nesterets, Y., Ternovski, D., Thompson, D., Wilkins, S. W., Stevenson, A. W., Sakellariou, A. & Taylor, J. A. (2011). *Proc. SPIE*, **8141**, 81410B.

[bb5] Maia, F. R. N. C. (2012). *Nat. Methods*, **9**, 854–855. 10.1038/nmeth.2110 22936162

[bb6] Morgan, A. J., Murray, K. T., Quiney, H. M., Bajt, S. & Chapman, H. N. (2020). *J. Appl. Cryst.* **53**. Submitted. 10.1107/S1600576720011991 PMC7710491 33304226

[bb7] Morgan, A. J., Prasciolu, M., Andrejczuk, A., Krzywinski, J., Meents, A., Pennicard, D., Graafsma, H., Barty, A., Bean, R. J., Barthelmess, M., Oberthuer, D., Yefanov, O. M., Aquila, A., Chapman, H. N. & Bajt, S. (2015). *Sci. Rep.* **5**, 9892. 10.1038/srep09892 PMC4450759 26030003

[bb8] Morgan, A. J., Quiney, H. M., Bajt, S. & Chapman, H. N. (2020). *J. Appl. Cryst.* **53**, 760–780. 10.1107/S1600576720005567 PMC7312131 32684891

[bb9] Morgan, K. S., Paganin, D. M. & Siu, K. K. W. (2012). *Appl. Phys. Lett.* **100**, 124102.

[bb10] Murray, K. T., Pedersen, A. F., Mohacsi, I., Detlefs, C., Morgan, A. J., Prasciolu, M., Yildirim, C., Simons, H., Jakobsen, A. C., Chapman, H. N., Poulsen, H. F. & Bajt, S. (2019). *Opt. Express*, **27**, 7120. 10.1364/OE.27.007120 30876283

[bb11] Nazaretski, E., Huang, X., Yan, H., Lauer, K., Conley, R., Bouet, N., Zhou, J., Xu, W., Eom, D., Legnini, D., Harder, R., Lin, C. H., Chen, Y. S., Hwu, Y. & Chu, Y. S. (2014). *Rev. Sci. Instrum.* **85**, 033707. 10.1063/1.4868968 24689592

[bb12] Nazaretski, E., Yan, H., Lauer, K., Bouet, N., Huang, X., Xu, W., Zhou, J., Shu, D., Hwu, Y. & Chu, Y. S. (2017). *J. Synchrotron Rad.* **24**, 1113–1119. 10.1107/S1600577517011183 29091054

[bb13] Paganin, D. M., Labriet, H., Brun, E. & Bérujon, S. (2018). *Phys. Rev. A*, **98**, 053813.

[bb14] Pelz, P. M., Guizar-Sicairos, M., Thibault, P., Johnson, I., Holler, M. & Menzel, A. (2014). *Appl. Phys. Lett.* **105**, 251101.

[bb15] Prasciolu, M., Leontowich, A. F. G., Krzywinski, J., Andrejczuk, A., Chapman, H. N. & Bajt, S. (2015). *Opt. Mater. Expr.* **5**, 748.

[bb16] Rodenburg, J. M., Hurst, A. C. & Cullis, A. G. (2007). *Ultramicroscopy*, **107**, 227–231. 10.1016/j.ultramic.2006.07.007 16959428

[bb17] Rohou, A. & Grigorieff, N. (2015). *J. Struct. Biol.* **192**, 216–221. 10.1016/j.jsb.2015.08.008 PMC6760662 26278980

[bb18] Thibault, P., Dierolf, M., Bunk, O., Menzel, A. & Pfeiffer, F. (2009). *Ultramicroscopy*, **109**, 338–343. 10.1016/j.ultramic.2008.12.011 19201540

[bb19] Thibault, P. & Menzel, A. (2013). *Nature*, **494**, 68–71. 10.1038/nature11806 23389541

[bb20] Yan, H., Conley, R., Bouet, N. & Chu, Y. S. (2014). *J. Phys. D Appl. Phys.* **47**, 263001.

[bb21] Zdora, M.-C. (2018). *J. Imaging*, **4**, 60.

[bb22] Zdora, M. C., Thibault, P., Zhou, T., Koch, F. J., Romell, J., Sala, S., Last, A., Rau, C. & Zanette, I. (2017). *Phys. Rev. Lett.* **118**, 203903. 10.1103/PhysRevLett.118.203903 28581800

